# Post-Synthesis Modification of Photoluminescent and Electrochemiluminescent Au Nanoclusters with Dopamine

**DOI:** 10.3390/nano11010046

**Published:** 2020-12-27

**Authors:** Jae Hyun Kim, Joohoon Kim

**Affiliations:** 1 Department of Chemistry, Research Institute for Basic Science, Kyung Hee University, Seoul 02447, Korea; blangka12@khu.ac.kr; 2 KHU-KIST Department of Converging Science and Technology, Kyung Hee University, Seoul 02447, Korea

**Keywords:** post-synthesis modification, Au nanoclusters, dopamine, photoluminescence, electrochemiluminescence

## Abstract

Here, we report a post-synthesis functionalization of the shell of Au nanoclusters (NCs) synthesized using glutathione as a thiolate ligand. The as-synthesized Au NCs are subjected to the post-synthesis functionalization via amidic coupling of dopamine on the cluster shell to tailor photoluminescence (PL) and electrochemiluminescence (ECL) features of the Au NCs. Because the NCs’ PL at *ca*. 610 nm is primarily ascribed to the Au(I)-thiolate (SG) motifs on the cluster shell of the NCs, the post-synthesis functionalization of the cluster shell enhanced the PL intensity of the Au NCs via rigidification of the cluster shell. In contrast to the PL enhancement, the post-synthesis modification of the cluster shell does not enhance the near-infrared (NIR) ECL of the NCs because the NIR ECL at *ca*. 800 nm is ascribed to the Au(0)-SG motifs in the metallic core of the NCs.

## 1. Introduction

Au nanoclusters (Au NCs) comprising a small number of atoms, typically with a particle size of ~1 nm, have attracted intense research in both fundamental and applied disciplines as a promising class of functional nanomaterials [1,2,3]. Recently, Au NCs have thus risen to the forefront of sensing, photovoltaic, and diagnostic research [4,5,6,7,8]. This is because the ultra-small Au NCs exhibit unique molecular-like behaviors, such as photoluminescence (PL), molecular chirality, quantized double-layer charging, and electrochemiluminescence (ECL), distinctively different from their larger nanoparticle and bulk counterparts [9,10,11,12]. These distinctive properties were found to be highly dependent on the size, oxidation states, composition, and ligands of the Au NCs [13,14,15,16].

A variety of ligands, such as alkanethiols, dendrimers, peptides, and proteins, have been used to synthesize well-defined Au NCs. Among the various ligands studied so far, a hydrophilic tripeptide glutathione (GSH) has been particularly useful for synthesizing Au NCs in aqueous medium with rational tuning of their properties [17,18]. For example, Negishi et al. synthesized a series of GSH-protected Au NCs ranging from Au_10_(SG)_10_ to Au_39_(SG)_24_ [19]. The GSH-protected Au NCs exhibited PL in the visible to near-infrared (NIR) region [20,21,22], and PL quantum yield of 8% was observed for Au_22_(SG)_18_ in water [20]. In addition, GSH-protected Au NCs were reported to exhibit intense ECL with an ECL quantum yield as high as 0.42% [23]. To tailor the photophysical/electrochemical properties of Au NCs in rational ways, various surface engineering approaches have been suggested, such as rigidification of the cluster shell, exchange of surface ligands on the cluster shell, surface functionalization of the cluster shell with light-harvesting ligands, and self-assembly of Au NCs through their ligand shells [24,25]. For example, rigidification of Au(I)-thiolate shell of Au NCs was proposed as a strategy to amplify the PL efficiencies of Au NCs [21,26], and ligand exchange was demonstrated as a methodology to enhance PL quantum yield of Au NCs stabilized by phenylethanethiolate ligands upon the ligand exchange with 1,4-dithiol durene [27]. Because brightness of Au NCs depends on the absorption efficiency of excitation light as well as the PL quantum yield of the NCs, light-harvesting ligands (i.e., chromophores) were introduced on the cluster shell of Au NCs to efficiently adsorb excitation light and to transfer the excitation energy from the surface-bound chromophores to the emitting Au NCs [28]. More recently, cationic polymer-mediated self-assembly of Au NCs was demonstrated for preparing a highly luminescent assembly of Au NCs depending on NCs’ surface ligands and cationic polymers [29].

Here, we report a post-synthesis functionalization of the shell of Au NCs with dopamine to investigate how the surface functionalization of Au NCs affects both PL and ECL of the Au NCs. The Au NCs were synthesized using GSH acting as a thiolate ligand, similar to previous reports [18,30]. In agreement with our recent studies, the as-synthesized Au NCs exhibited a strong NIR ECL peak with a shoulder peak in the visible region, while the nanoclusters emitted a strong PL peak primarily in the visible region [30,31]. We ascribed the NIR and visible emission peaks of the nanoclusters to the Au(0)-SG motif in their metallic core and the Au(I)-SG motif on their shell surface, respectively [30,31]. The as-synthesized Au NCs were subjected to the post-synthesis functionalization for surface coupling of dopamine on the cluster shell. Spectroscopic measurements including ultraviolet-visible (UV-vis) absorption, Fourier-transform infrared (FT-IR), and nuclear magnetic resonance (NMR) confirmed the successful coupling of dopamine. Interestingly, the surface functionalization enhanced the PL intensity of the Au NCs but weakened the NIR ECL of the clusters. This finding is important because it demonstrates the significance of surface-engineered Au NCs to tailor the clusters’ PL and ECL as a post-synthesis functionalization of the cluster shell.

## 2. Materials and Methods

### 2.1. Materials

All chemicals were used as received without further purification. The following chemicals were received from Sigma Aldrich, Inc. (St. Louis, MO, USA): gold(III) chloride trihydrate (HAuCl_4_⋅3H_2_O, ≥99.9%, trace metals basis), L-glutathione reduced (GSH, ≥98.0%), 9-fluorenylmethoxycarbonyl chloride (Fmoc chloride, 97%), tetrahydrofuran (THF, ≥99.9%, anhydrous), dopamine hydrochloride, potassium nitrate (KNO_3_, 99.999%), phosphate-buffered saline (PBS) buffer, 1-[3-(dimethylamino)propyl]-3-ethylcarbodiimide methiodide (EDC), *N*-hydroxy-succinimide (NHS, 98%), triethylamine (TEA, ≥99%), gold standard for ICP (1000 mg/L Au in HCl), and deuterium oxide (D_2_O, 99.9 atom % D). Sodium bicarbonate (NaHCO_3_, ≥99.0%) was obtained from Daejung Inc. (Gyeonggi, Korea). Hydrochloric acid (HCl, 35.0~37.0%) and nitric acid (HNO_3_, 60.0%) were purchased from Samchun Chemical Co., Ltd. (Seoul, Korea). Amicon^®^ Ultra-4 centrifugal ultrafiltration filters (3 kDa MWCO) were purchased from Merck Millipore Corp. (Danvers, MA, USA). Deionized water (DI water, 18 MΩ⋅cm) was used in the preparation of aqueous solutions (Aquapuri541, YOUNG IN Chromass, Gyeonggi, Korea).

### 2.2. Synthesis and Post-Synthesis Modification of NCs

Au NCs were synthesized using GSH as both a reductant and stabilizer by following a previously reported procedure [18,30]. In brief, fresh aqueous solutions of 100 mM GSH (0.6 mL) and 20 mM HAuCl_4_⋅3H_2_O (2.0 mL) were added to 17.4 mL of DI water under stirring. The aqueous mixture was kept under vigorous stirring at room temperature for 1 min and subsequently heated to 70 °C for 24 h. After following this synthesis procedure, the as-synthesized Au NCs were subjected to a post-synthesis modification as follows: 180 mmol of Fmoc chloride was dissolved in 1.0 mL of THF containing 1 mg of NaHCO_3_. This solution was added to 1.0 mL of the as-synthesized Au NCs (10 mg/mL) in DI water. After the solution was stirred vigorously at room temperature for 3 h, 8.0 mL of THF was added to the mixture, followed by centrifugation at 3600 rpm for 15 min to precipitate Fmoc-protected Au NCs that had formed. The collected Fmoc-protected Au NCs were washed twice with THF and then dried overnight, after which 10 mg of purified Fmoc-protected Au NCs were re-dissolved in 2.0 mL of PBS buffer (pH 7.4). Then, 60 mmol each of EDC and NHS were added to 2.0 mL of Fmoc-protected Au NCs (5.0 mg/mL) and kept under stirring at room temperature for 20 min; then, 120 mmol of dopamine hydrochloride dissolved in DI water was added. After the mixture was stirred for 16 h, the cluster product was purified with centrifugal ultrafiltration filters (3 kDa MWCO), resulting in dopamine-conjugated Au NCs.

### 2.3. Characterization

UV-vis absorption and PL spectra were recorded using an Agilent 8453 UV-vis spectrometer (Agilent Tech., Wilmington, DE, USA) and a FS-2 fluorescence spectrometer (Scinco Co., Seoul, Korea), respectively. Absolute PL quantum yields were determined using a Quantaurus-QY C11347 spectrometer (Hamamatsu Photonics K.K., Shizuoka, Japan). Inductively coupled plasma-atomic emission spectroscopy (ICP-AES) was carried out to determine the GSH-to-Au ratio of clusters using an iCAP^TM^ 7200 spectrometer (Thermo Scientific, Waltham, MA, USA). Transmission electron microscopy (TEM) was performed using a Tecnai G^2^ F30 S-Twin instrument (FEI Co., Hillsboro, OR, USA) operating at 200 kV. TEM samples were prepared by dropping aqueous cluster solutions onto 200 mesh carbon-coated Ni grids (Ted Pella Inc., Redding, CA, USA) followed by drying in air. NMR spectroscopy was carried out with an Avance III HD 400 spectrometer (Bruker Corp., Billerica, MA, USA). NMR samples were prepared by dissolving clusters in D_2_O. FT-IR spectroscopy was conducted on an Alpha II spectrometer (Bruker Corp., Billerica, MA, USA). Dynamic light scattering (DLS) measurements were performed with a Zetasizer Nano ZS90 molecular analyzer (Malvern Panalytical, Malvern, UK). ECL spectra were recorded using a three-electrode electrochemical cell connected to the slit of an Acton Standard SP2150 monochromator (Princeton Instruments, Trenton, NJ, USA) equipped with a PIXIS 100B charge-coupled device (CCD) camera (Princeton Instruments, Trenton, NJ, USA). For all electrochemical measurements, we used a CHI440A potentiostat (CH Instruments Inc., Bee Cave, TX, USA) with the three-electrode cell, and we used a glassy carbon electrode (dia.: 3 mm) as the working electrode. We used an Ag/AgCl (3 M NaCl) and a coiled Pt wire as the reference electrode and the auxiliary electrode, respectively.

## 3. Results and Discussion

**Figure 1:**
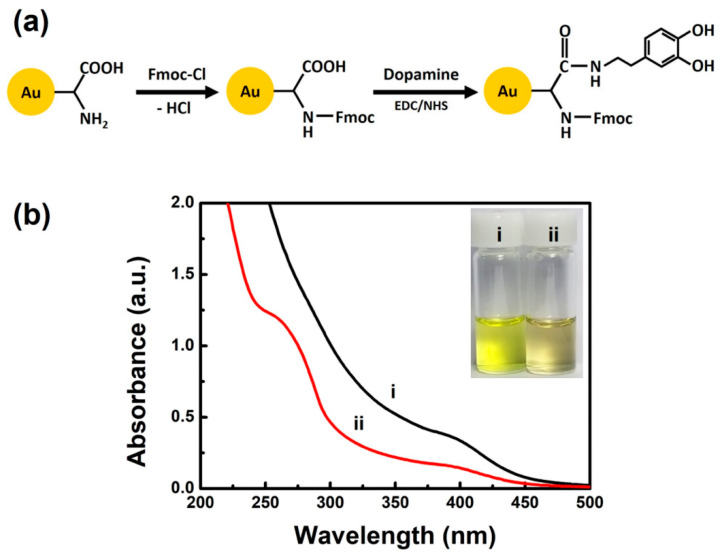
(**a**) Schematic illustration of post-synthesis functionalization of as-synthesized Au NCs with dopamine. (**b**) UV-vis absorption spectra of (i) as-synthesized Au NCs and (ii) dopamine-conjugated Au NCs. The inset shows a digital photo of as-synthesized Au NCs and dopamine-conjugated Au NCs in water under visible light. Concentration of Au NCs: 1.5 mg/mL.

As shown in Figure 1a, we protected the amino groups of GSH on the shell of the as-synthesized Au NCs with Fmoc chloride. The resulting Fmoc-protected Au NCs were then functionalized via covalent coupling of dopamine to carboxyl groups of GSH on the cluster shell (see Materials and Methods Section for details). Figure 1b shows UV-vis absorption spectra of the as-synthesized Au NCs and the dopamine-conjugated Au NCs. The dopamine-conjugated Au NCs were pale yellow in aqueous solution, while the as-synthesized Au NCs were bright yellow (inset in Figure 1b). The as-synthesized Au NCs ((i) in Figure 1b) exhibited a broad UV-vis absorption peak at *ca*. 400 nm but did not show the surface plasmon absorption band typically observed for Au nanoparticles larger than 2 nm [32]. These UV-vis absorption characteristics agreed with those observed for small GSH-protected Au NCs that emit orange PL with a maximum peak at *ca*. 610 nm via aggregation-induced emission (AIE) of Au(I)-SG motif in the compact shell of the NCs [18]. The content of GSH in the as-synthesized Au NCs was quantified by determining the GSH-to-Au ratio in the clusters. ICP-AES analysis revealed that GSH contributed 54% of the as-synthesized Au NCs by weight. This result could be translated to a GSH-to-Au ratio of 0.76:1, which was consistent with the value (i.e., 0.84:1) previously reported by thermal gravimetric analysis (TGA) of the GSH-protected Au NCs [18]. For the dopamine-conjugated Au NCs ((ii) in Figure 1b), we observed a new UV-vis absorption shoulder peak at *ca*. 270 nm, which is similar to that of dopamine (Supplementary Materials, Figure S1), thus indicating successful surface coupling of dopamine on the shell surface of NCs.

**Figure 2:**
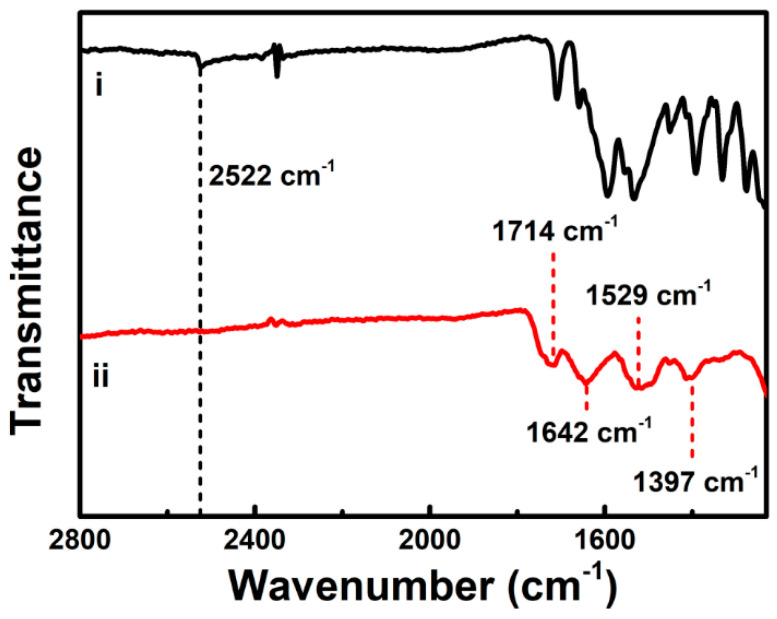
FT-IR spectra of (i) free GSH and (ii) as-synthesized Au NCs in the 1000–2800 cm^−1^ region.

**Figure 3:**
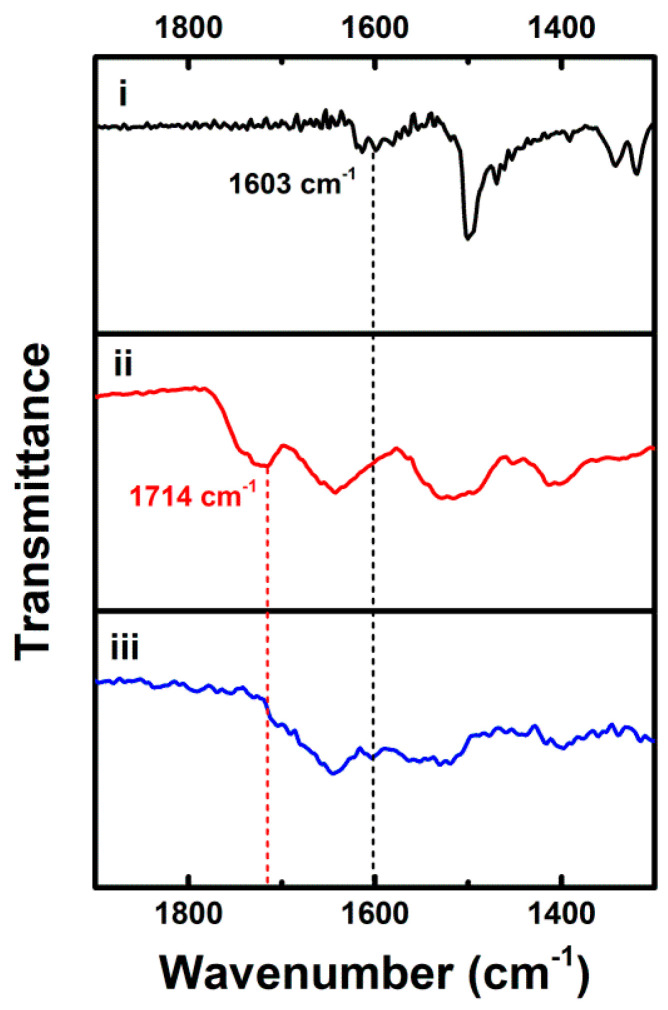
FT-IR spectra of (i) free dopamine, (ii) as-synthesized Au NCs, and (iii) dopamine-conjugated Au NCs in the 1300–1900 cm^−1^ region.

In addition to the UV-vis absorption measurements, we performed FT-IR and NMR studies to confirm the covalent coupling of dopamine to carboxyl groups of GSH on the cluster shell. Figure 2 shows FT-IR spectra of free GSH and the as-synthesized Au NCs. Unlike the free GSH counterpart ((i) in Figure 2), the as-synthesized Au NCs did not show a band at *ca*. 2522 cm^−1^, corresponding to S–H stretching vibration mode, in their FT-IR spectrum ((ii) in Figure 2) [33]. The disappearance of the FT-IR band at 2522 cm^−1^ in the as-synthesized Au NCs indicates the adsorption of GSH in the form of the thiolate (GS) to the Au surface via the Au-S bond formation, as reported previously [19]. Other characteristic FT-IR bands of the as-synthesized Au NCs appeared wider but otherwise roughly the same as those in the FT-IR spectrum of the free GSH, which further suggests GSH ligation to the Au surface. The main characteristic bands are the absorption bands of C=O stretching mode at *ca.* 1714 cm^−1^, amide I mode (mainly C=O stretching) at *ca*. 1642 cm^−1^, amide II mode (mainly N–H bending and C–N stretching) at *ca*. 1529 cm^−1^, and C–O stretching mode at *ca*. 1397 cm^−1^. All of these bands indicate the presence of GSH adsorbed on the Au surface. The as-synthesized Au NCs were subjected to post-synthesis modification for the covalent coupling of dopamine via amidic coupling to carboxyl groups on the cluster shell. Compared with the FT-IR spectrum of the as-synthesized Au NCs ((ii) in Figure 3), the prominent band at *ca*. 1714 cm^−1^, attributed to the C=O stretching band of carboxyl moieties of GSH, vanished upon the covalent coupling of dopamine to the surface carboxyl groups ((iii) in Figure 3), which indicates the covalent conjugation of dopamine on the cluster shell after the post-synthesis modification. We also verified the presence of conjugated dopamine in the FT-IR spectrum of the dopamine-conjugated Au NCs with the appearance of FT-IR bands at *ca*. 1603 cm^−1^ corresponding to C–C bonds in the aromatic ring of dopamine ((i) and (iii) in Figure 3). Furthermore, ^1^H NMR spectrum of the as-synthesized Au NCs shows major peaks corresponding to methylene groups of GSH (Supplementary Materials, Figure S2a), indicating the passivation of the clusters with GSH, as reported previously [26]. More specifically, by comparison with previous NMR studies of free GSH and GSH-stabilized Au NCs [34,35], the merged broad peaks around ~3.9 ppm were assigned to the CH_2_ at C-9 and CH at C-1, as shown in Figure S2a (Supplementary Materials). The broad multiplets at 2.1 and 2.5 ppm were also assigned to the CH_2_ at C-2 and C-3, respectively. Note that the minor peaks at 2.8–3.2 ppm and 2.3 ppm were from residual glutathione disulfide (GS-SG), as reported previously [36]. In addition, the ^1^H NMR spectrum of the dopamine-conjugated Au NCs shows peaks related to the aromatic and aliphatic protons of dopamine (Supplementary Materials, Figure S2b), verifying the surface coupling of dopamine on the cluster shell after the post-synthesis functionalization. The ^1^H NMR spectrum also displays no detectable peaks related to dihydroxyindole (DHI), known as a key precursor involved in the oxidative polymerization of dopamine to polydopamine, indicating the absence of polydopamine in the dopamine-conjugated Au NCs [37,38]. The integration of peak intensities in the ^1^H NMR spectrum of the dopamine-conjugated Au NCs provides the estimated molar ratio of coupled dopamine to GSH (i.e., ~0.8) on the cluster shell.

**Figure 4:**
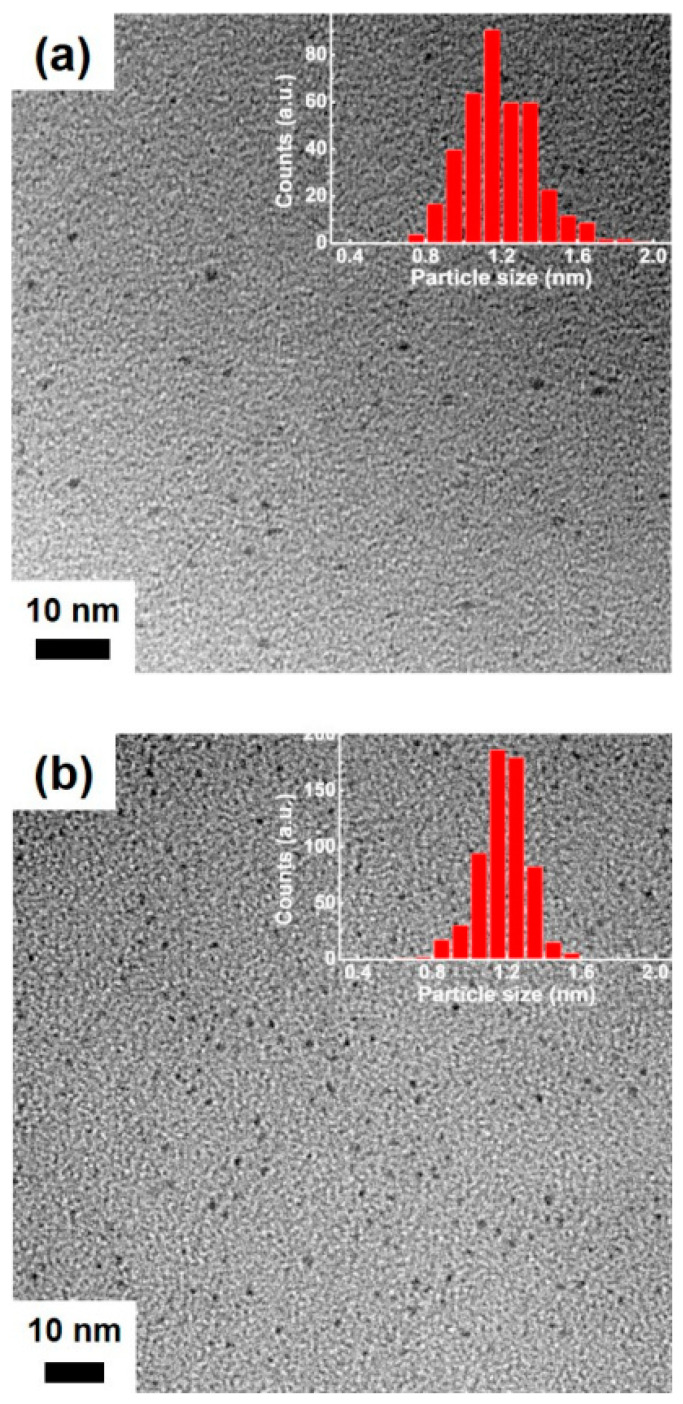
TEM images of (**a**) as-synthesized Au NCs and (**b**) dopamine-conjugated Au NCs. The insets of (**a**) and (**b**) show size distribution histograms of the NCs.

Figure 4a shows a TEM image of the as-synthesized Au NCs, indicating that the NCs were ultra-small and fairly uniform in size, as reported previously for Au NCs synthesized using GSH [18,39]. The size distribution histogram of the NCs revealed a narrow size distribution of the Au metal core with an average size of 1.2 ± 0.2 nm (inset in Figure 4a). The measured small size of the as-synthesized Au NCs (i.e., less than 2 nm) was also supported by the absence of the Au surface plasmon absorption band in the UV-vis absorption spectrum of the NCs, as discussed earlier ((i) in Figure 1b). Interestingly, as shown in Figure 4b, the measured size of the dopamine-conjugated Au NCs (i.e., 1.2 ± 0.1 nm) did not differ significantly from that of the as-synthesized Au NCs, which indicates no significant aggregation of the NCs even after the covalent conjugation of dopamine on the cluster shell. In contrast, DLS measurements of both the as-synthesized and the dopamine-conjugated Au NCs revealed a significant change in the clusters’ hydrodynamic diameters from 1.8 ± 1.2 nm for the as-synthesized Au NCs to 627.1 ± 147.9 nm for the dopamine-conjugated Au NCs. Because little aggregation of Au metal core occurred in the clusters (Figure 4), this substantial change in their hydrodynamic diameters was primarily attributed to the dopamine conjugation to GSH on the cluster shell [40]. These results indicate that the post-synthesis functionalization of the as-synthesized Au NCs led to successful coupling of dopamine on the cluster shell, while keeping the NCs from aggregating.

**Figure 5:**
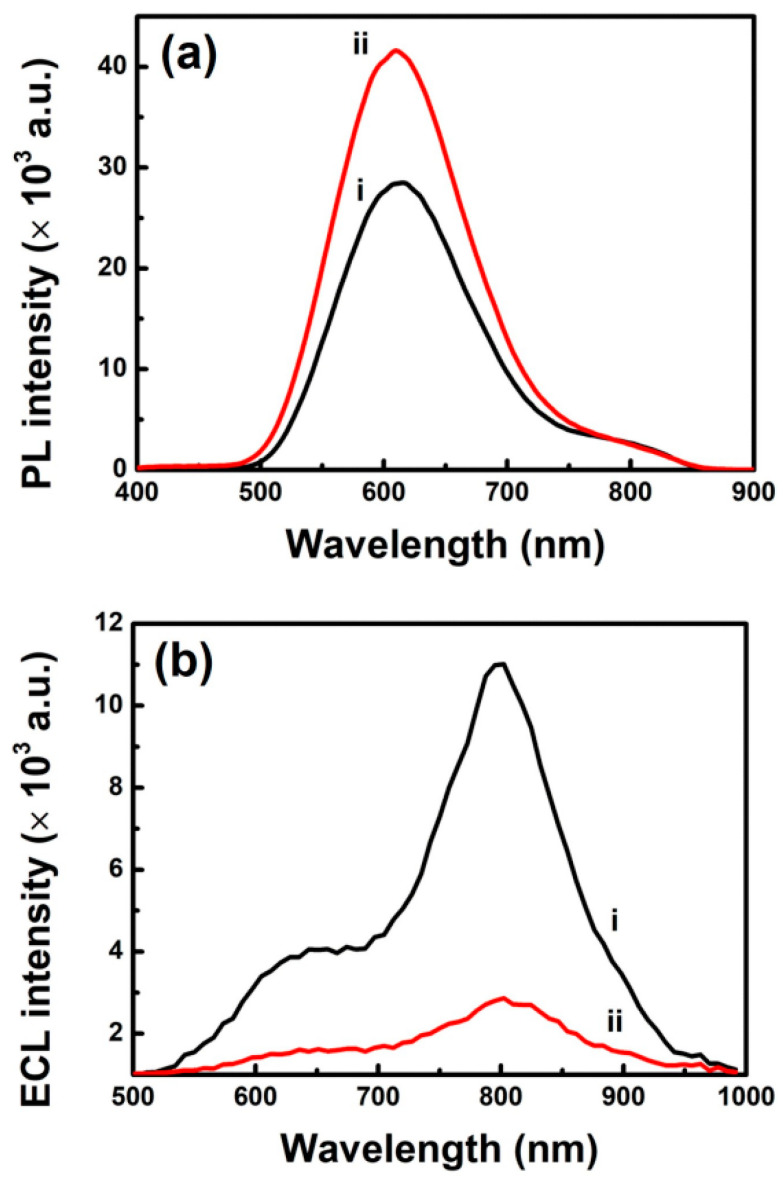
(**a**) PL emission spectra of (i) as-synthesized Au NCs and (ii) dopamine-conjugated Au NCs. λ_ex_ = 365 nm. Concentration of Au NCs: 1.5 mg/mL. (**b**) ECL spectra of (i) as-synthesized Au NCs and (ii) dopamine-conjugated Au NCs in the presence of 0.5 M TEA as a co-reactant. Electrolyte: 0.1 M KNO_3_. Applied potential: 1.3 V. Concentration of Au NCs: 0.75 mg/mL.

We measured PL and ECL to investigate the influence of the post-synthesis functionalization of the Au NC shells with dopamine on the clusters’ photophysical and electrochemical properties. Figure 5a shows PL spectra of the as-synthesized Au NCs and the dopamine-conjugated Au NCs. The as-synthesized Au NCs exhibited orange PL with a maximum peak at *ca*. 610 nm, with a weak shoulder peak at *ca*. 790 nm upon excitation at λ_ex_ = 365 nm ((i) in Figure 5a). The orange PL of the as-synthesized Au NCs at 610 nm was in agreement with the PL emission observed for the Au NCs synthesized using GSH in previous reports, wherein the PL was attributed to AIE of the Au(I)-SG motifs on their shell surface [18,30,31]. The PL emission could be ascribed to ligand-to-metal charge transfer (LMCT) or ligand-to-metal-metal charge transfer (LMMCT) from the sulfur atom to the Au atoms in the dense Au(I)-SG shell on the cluster surface, and subsequent radiative relaxation [18,41]. After the post-synthesis functionalization of the as-synthesized Au NCs with dopamine, we observed the increase in PL intensity of the clusters, especially at 610 nm ((ii) in Figure 5a). Because the orange PL at 610 nm was ascribed to the Au(I)-SG motifs on the NC surface, the observed increase in PL intensity of the dopamine-conjugated Au NCs suggested dopamine conjugation on the NC surface via the post-synthesis functionalization of the NCs. The PL enhancement of the dopamine-conjugated Au NCs primarily at 610 nm also suggested that the electron-donating capability of the ligands on the dopamine-conjugated Au NCs did not play a major role in the PL enhancement of the clusters despite the possible ligand to metal nanoparticle core charge transfer (LMNCT) previously reported [16]. This is most likely because the orange PL at 610 nm was attributed to AIE of Au(I)-SG motif in the shell of the NCs, as discussed earlier. By integrating PL intensities of the clusters over the wavelength, we observed an increase of 1.5 times in the PL emission of the dopamine-conjugated Au NCs over that of the as-synthesized clusters. In addition, the PL quantum yields of the as-synthesized Au NCs and the dopamine-conjugated Au NCs were found to be 1.9% and 2.6%, respectively. In recent studies, investigators suggested rigidification of the Au(I)-thiolate shell of Au NCs as an effective strategy to enhance the PL of Au NCs [21,26,42]. For example, Lee and coworkers demonstrated PL enhancement of GSH-protected Au NCs (i.e., Au_22_(SG)_18_) by rigidifying the shells of NCs with lipophilic bulky tetraoctylammonium cations [21]. More recently, a similar PL enhancement was reported by rigidifying the shell of Au_22_(SG)_18_ via covalent modification of the SG ligand shells on the clusters with aromatic pyrene chromophores [26]. Therefore, we attributed the observed PL enhancement for the dopamine-conjugated Au NCs to shell rigidification that arose from the π–π interactions between aromatic catechol moieties of dopamine conjugated on the cluster shell. It is noteworthy that the PL enhancement for the dopamine-conjugated Au NCs might be attributable to AIE caused by the dopamine conjugation on the NCs [43,44,45]. However, we ruled out the possibility of PL enhancement by AIE because TEM measurements of the dopamine-conjugated Au NCs showed little aggregation of the NCs, as discussed earlier. In addition to the PL emission spectra, we measured PL excitation spectra of the as-synthesized Au NCs and the dopamine-conjugated Au NCs (Supplementary Materials, Figure S3). Figure S3 shows the PL excitation spectra displaying a maximum around *ca*. 400 nm, which coincided with the broad absorption shoulder observed in the UV-vis absorption spectra of the NCs (Figure 1b). Meanwhile, Figure 5b shows ECL spectra of the as-synthesized Au NCs and the dopamine-conjugated Au NCs. Their ECL spectra were obtained at a potential of 1.3 V in the presence of TEA as an anodic co-reactant for oxidative-reduction ECL reactions. In agreement with previous studies [30,31], the ECL spectra show a strong NIR ECL peak at *ca*. 800 nm with a shoulder peak at *ca*. 620 nm. Because the shoulder peak wavelength matched the clusters’ orange PL, we attributed the shoulder ECL emission to the Au(I)-SG motifs on the NC surface, as reported in the previous findings [30,31]. Similarly, the NIR ECL emission was ascribed to the Au(0)-SG motifs, responsible for the PL of the clusters at 790 nm, in the metallic core of the Au NCs [30,31]. Feasible electrochemical oxidation of the Au(0)-SG motifs, in contrast with that of the Au(I)-SG motifs, was responsible for the clusters’ strong NIR ECL emission. In contrast to the PL enhancement, after functionalizing the cluster shell of the as-synthesized Au NCs with dopamine, we observed decreased ECL intensity of the clusters. Because the Au(0)-SG motifs in the cluster core were responsible for their NIR ECL, the diminished ECL emission of the dopamine-conjugated Au NCs suggested that the possible LMNCT previously reported did not play a significant role in the ECL enhancement of the clusters, as we discussed for their PL enhancement earlier [16]. Instead, the dopamine-conjugated Au NCs showed diminished ECL emission ((ii) in Figure 5b), presumably attributable to deteriorated electrical conductivity, which limited the electrochemical oxidation required for anodic ECL generation of the NCs with the organic dopamine conjugated on the cluster surface.

## 4. Conclusions

In summary, here, we reported a post-synthesis modification of the shell of Au NCs with dopamine for tailoring PL and ECL of the NCs. The Au NCs were synthesized using GSH as a thiolate ligand and then subjected to the post-synthesis functionalization via amidic coupling of dopamine on the cluster shell. The post-synthesis functionalization of the as-synthesized Au NCs led to an increase in the PL with a maximum peak at 610 nm (i.e., orange PL), but the clusters’ NIR ECL decreased. Combined with the findings from previous studies that the orange color PL and the NIR ECL of the Au NCs could be attributed to the Au(I)-SG motifs on the cluster shell and the Au(0)-SG motifs in the core of the NCs respectively [30,31], the present study could rationalize both the increase in the clusters’ orange PL and the decrease in their NIR ECL that we observed after we functionalized the cluster shell of the as-synthesized Au NCs with dopamine. The findings of the present study demonstrate the significance of the post-synthesis functionalization of the cluster shell as surface engineering of NCs to tailor the clusters’ PL and ECL features. Furthermore, because the post-synthesis modification of the shell of Au NCs is a feasible and facile functionalization strategy of Au NCs with little dependency on any synthetic methods of the Au NCs, we envision that it can be applied to many different GSH-protected Au NCs for tailoring PL and ECL features of the clusters.

## Data Availability

The data presented in this study are available on request from the corresponding author. The data are not publicly available due to institutional and funding regulations.

## Supplementary Materials

The following are available online at https://www.mdpi.com/2079-4991/11/1/46/s1, Figure S1: UV-vis absorption spectrum of free dopamine, Figure S2: ^1^H NMR spectra of (a) as-synthesized Au NCs and (b) dopamine-conjugated Au NCs. Figure S3: PL excitation spectra of (i) as-synthesized Au NCs and (ii) dopamine-conjugated Au NCs.
