# Supplementary material for: Post-Synthesis Modification of Photoluminescent and Electrochemiluminescent Au Nanoclusters with Dopamine
Source: Nanomaterials (Basel). 2020 Dec 27;11(1):46. doi: 10.3390/nano11010046 (PMC7824466; doi:10.3390/nano11010046)
Supplement: Supplementary file 1 [file nanomaterials-11-00046-s001.pdf]

Supporting Information

Post-synthesis Modification of Photoluminescent and Electrochemiluminescent Au Nanoclusters with Dopamine

Jae Hyun Kim [1] and Joohoon Kim [1,2,*]

[1] Department of Chemistry, Research Institute for Basic Sciences, Kyung Hee University, Seoul 02447, Republic of Korea

[2] KHU-KIST Department of Converging Science and Technology, Kyung Hee University, Seoul 02447, Republic of Korea

***Corresponding Author: jkim94@khu.ac.kr (J. Kim)**

Table of Contents:

Supporting Figures S1-S3 S2 – S4

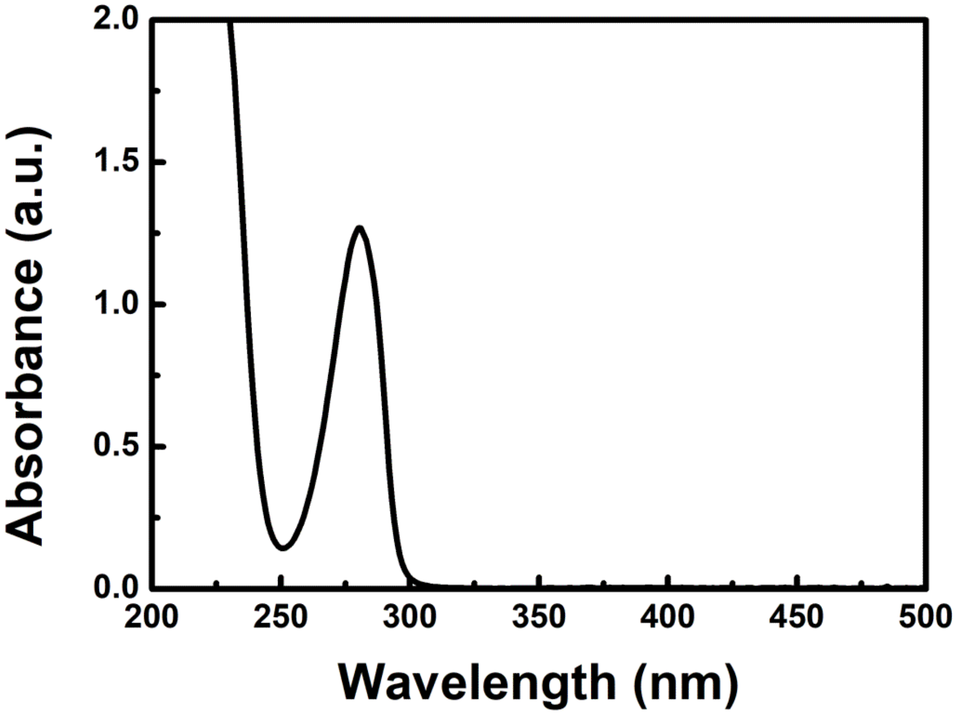

Figure S1. UV-vis absorption spectrum of free dopamine.

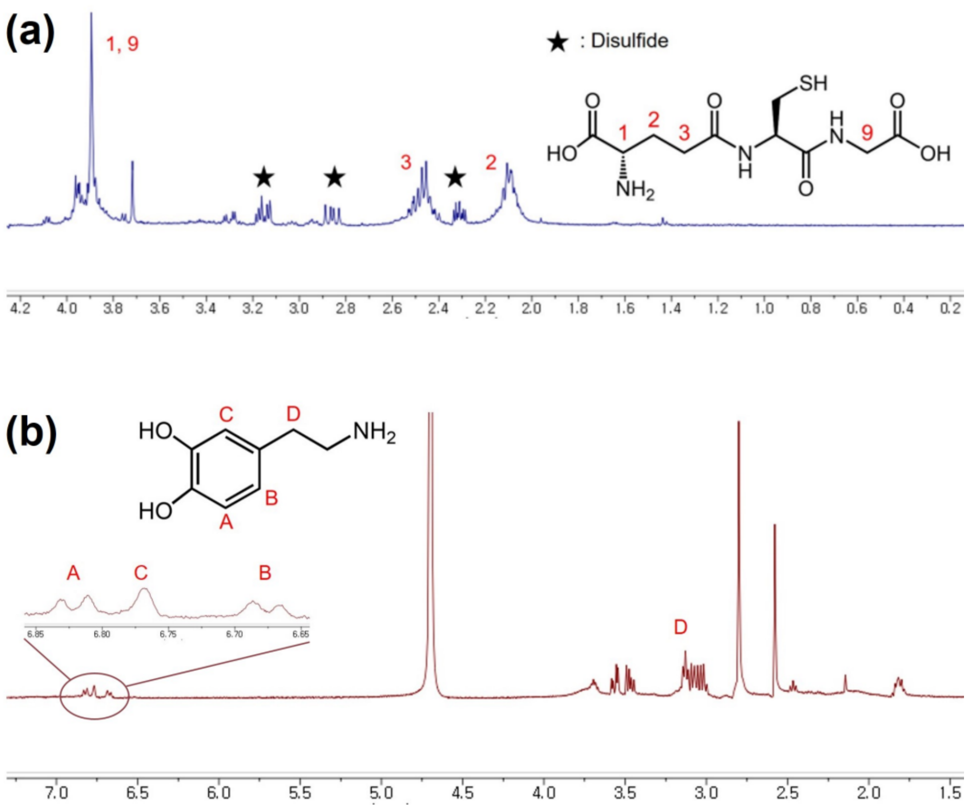

Figure S2. ^{1}H NMR spectra of (a) as-synthesized Au NCs and (b) dopamine-conjugated Au NCs. NMR samples were prepared by dissolving clusters in D_2O.

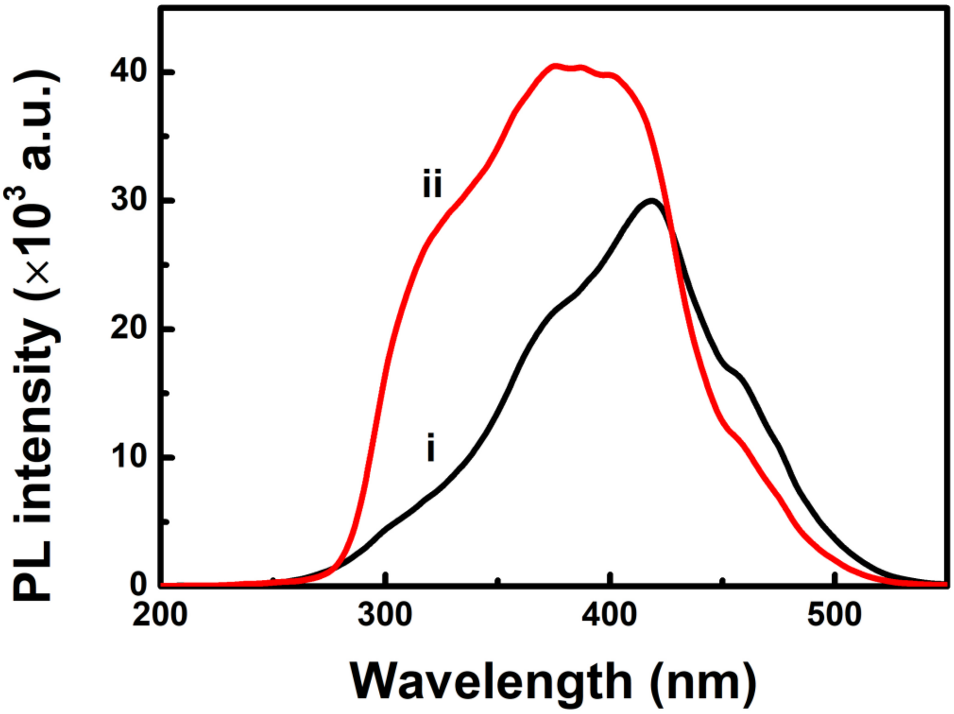

Figure S3. PL excitation spectra of (i) as-synthesized Au NCs and (ii) dopamine-conjugated Au NCs. λ_{em} = 610 nm.
